# Estradiol Inhibits ER Stress-Induced Apoptosis in Chondrocytes and Contributes to a Reduced Osteoarthritic Cartilage Degeneration in Female Mice

**DOI:** 10.3389/fcell.2022.913118

**Published:** 2022-05-20

**Authors:** Rita Dreier, Thomas Ising, Markus Ramroth, Yvonne Rellmann

**Affiliations:** Institute of Physiological Chemistry and Pathobiochemistry, University of Muenster, Muenster, Germany

**Keywords:** estradiol, cartilage, er stress, osteoarthritis, apoptosis

## Abstract

Gender differences are a common finding in osteoarthritis (OA). This may result from a differential response of males and females to endoplasmic reticulum (ER) stress in articular chondrocytes. We have previously described that ER stress in cartilage-specific ERp57 KO mice (ERp57 cKO) favors the development of knee OA, since this stress condition cannot be adequately compensated in articular chondrocytes with increasing age leading to the induction of apoptotic cell death and subsequent cartilage degeneration. The aim of this study was to enlighten gender-specific differences in ER stress, apoptosis, and OA development in ERp57 cKO mice. The analyses were extended by *in vitro* studies on the influence of estradiol in CRISPR/Cas9-generated C28/I2 ERp57 knock out (KO) and WT cells. ER stress was evaluated by immunofluorescence analysis of the ER stress markers calnexin (Cnx) and binding-immunoglobulin protein (BiP), also referred to as glucose-regulating protein 78 (GRP78) *in vivo* and *in vitro*. Apoptotic cell death was investigated by a commercially available cell death detection ELISA and TUNEL assay. OA development in mice was analyzed by toluidine blue staining of paraffin-embedded knee cartilage sections and quantified by OARSI-Scoring. Cell culture studies exhibited a reduction of ER stress and ER stress-induced apoptosis in C28/I2 cells in presence of physiological estradiol concentrations. This is consistent with a slower increase in age-related ER stress and a reduced number of apoptotic chondrocytes in female mice compared to male littermates contributing to a reduced osteoarthritic cartilage degeneration in female mice. Taken together, this study demonstrates that the female sex hormone estradiol can reduce ER stress and ER stress-induced apoptosis in articular chondrocytes, thus minimizing critical events favoring osteoarthritic cartilage degeneration. Therefore, the inhibition of ER stress through a modulation of effects induced by female sex hormones appears to be attractive for OA therapy.

## Introduction

OA is a chronic and progressive joint disease that results in severe loss of articular cartilage accompanied by subchondral bone remodeling, osteophyte formation, and synovial inflammation. It is the most common pathological condition of the musculoskeletal system worldwide and so in Germany, where half of all women and one third of all men over the age of 65 are affected (Fuchs, 2017).

Gender-specific differences and regulatory effects by sex hormones in OA were shown in several studies. For example, the incidence of OA in women vividly increases around the age of 50 when menopause starts, while there is a continuous increase of OA with age in men (Wilson et al., 1990). Furthermore, women tend to have a higher disease severity in knee OA especially after menopause (Srikanth et al., 2005). In addition, shorter estrogen exposure, e.g., through later age at menarche, shorter hormone replacement therapy, or shorter use of oral contraceptives, is associated with a higher risk of joint replacement arthroplasty of the knee due to severe knee OA (Eun et al., 2022). All these observations suggest a protective effect of estrogen in the development, progression or severity of OA, which should be investigated in further detail.

The exact molecular mechanisms of estradiol have not yet been described. However, OA development is associated with increased ER stress in articular chondrocytes, leading to apoptosis of cartilage cells (Nugent et al., 2009; Takada et al., 2011; Uehara et al., 2014; Hwang and Kim, 2015). Therefore, the influence of sex hormones on ER stress-induced chondrocyte apoptosis as a contributing factor in OA is the focus of this study.

In general, ER stress occurs when the capacity of the ER to fold proteins exceeds. ER stress is a consequence of increased loads of secretory proteins, hypoxia, oxidative stress, the presence of mutated proteins to be folded, or dysfunction or loss of chaperones (Rellmann et al., 2021a). *In vitro* ER stress often is induced with thapsigargin (Tg) lowering the levels of Ca^2+^ in the ER lumen. ER stress is likely involved in OA, since the OA onset is associated with an increased protein synthesis of both extracellular matrix (ECM) molecules and matrix-degrading proteases (Goldring and Goldring, 2007). This causes a high protein load in the ER of cartilage cells.

In such an ER stress situation hydrophobic stretches of misfolded proteins are recognized by the chaperone binding-immunoglobulin protein (BiP) also referred to as Glucose-regulating protein 78 (GRP78), which then interacts with the following three ER stress sensor proteins, located in the ER membrane: inositol-requiring protein 1 (IRE1), protein kinase RNA-like ER kinase (PERK) and activating transcription factor 6 (ATF6). These initiate the unfolded protein response (UPR) (Hetz et al., 2020). IRE1, PERK and ATF6 control adaptive processes in order to reduce or eliminate ER stress and to restore normal cell function. They initiate reduced translation, increased protein folding, ER biogenesis, improved protein entry to the ER, augmented ER-associated degradation (ERAD), autophagy and secretion, among others (Hetz, 2012). In chondrocytes, the UPR also changes proliferation and differentiation processes (Tsang et al., 2007). However, if UPR signaling fails to resolve the overload of misfolded proteins in the ER, cell death by apoptosis is induced to remove the stressed cells and protect the organism (Hetz et al., 2020). In late stages of OA, articular cartilage often becomes hypocellular and in the cartilaginous tissue empty lacunae become visible (Charlier et al., 2016). This high level of apoptotic chondrocytes is likely due to unresolvable ER stress.

It is known, that the lack of a specific protein disulfide isomerase, called ERp57 (or PDIA3), results in ER stress with UPR induction in chondrocytes *in vitro* and *in vivo* (Rellmann et al., 2021b). ERp57 is a member of the protein disulfide isomerase (PDI) family of ER resident chaperones, acts in a complex with calnexin (Cnx) or calreticulin (Calr) and therefore, is involved in disulfide bridge formation in glycoproteins (Coe and Michalak, 2010; Hettinghouse et al., 2018). Misfolding results in protein aggregation in the ER and affects growth plates and articular cartilage (Linz et al., 2015; Rellmann et al., 2021b).

To gain insight into the role of estradiol in ER stress-induced apoptosis of chondrocytes and the hormone’s contribution in reducing osteoarthritic cartilage degradation, here, gender-specific differences in ER stress, apoptosis, and OA development are analyzed in cartilage-specific ERp57 KO mice (cKO) (Linz et al., 2015). The investigations are extended by *in vitro* analyses elucidating the influence of estradiol on ER stress and apoptosis in CRISPR/Cas9-generated C28/I2 ERp57 knock out (KO) cells (Rellmann et al., 2019).

## Materials and Methods

### Culture of C28/I2 Cells

C28/I2 wildtype cells (WT) (Finger et al., 2003) were cultured in DMEM (Biochrom, Berlin, Germany) supplemented with 10% FCS, 1% sodium pyruvate, 100 units/ml penicillin, and 100 μg/ml streptomycin (DMEM complete) at 37°C, 5% CO2, and 100% humidity. Similar conditions were used for C28/I2 ERp57 knockout cells (KO), generated with CRISPR/Cas9 technology (Rellmann et al., 2019).

### Immunofluorescence Analysis of C28/I2 Cells

For immunofluorescence analysis, 5,000 C28/I2 WT and KO cells each were seeded in DMEM complete into wells of 8-well IBIDI slides with removable chambers (80,841, ibidi GmbH, Gräfelfing, Germany). After 4 h, medium was changed to fetal calf serum (FCS)-free DMEM complete, supplemented with 60 μg/ml β-aminopropionitrile fumarate, 25 μg/ml sodium ascorbate, 1 mM cysteine and 1 mM pyruvate (ABCP) +/- 10^–6^ M Tg, +/− 10^–11^ or 10^–15^ M estradiol. Tg is an inhibitor of the sarco/endoplasmic reticulum Ca^2+^-ATPase (SERCA), which creates low calcium levels in the ER inducing ER stress (Denmeade and Isaacs, 2005). After a culture period of 48 h at 37°C, 5% CO_2_ and 100% humidity, the cells were fixed in 1% paraformaldehyde (PFA) in Phosphate buffered saline (PBS) at pH 7.4 (PFA/PBS) for 10 min at room temperature (RT), washed twice for 5 min with PBS and then permeabilized with ethanol:acetic acid (2:1) for 5 min at −20 °C. Before immunofluorescence staining, the samples were washed with PBS (3 × 5 min) and unspecific protein binding was blocked for 1 h with 5% BSA in PBS at RT. To analyze ER stress, the cells were incubated overnight at 4 °C with anti-BiP antibody (ADI-SPA-768–0,050, Enzo Life Science, Farmingdale, NY, United States, 1:200) or anti-Cnx antibody (ADI-SPA-860D, Enzo Life Science, Farmingdale, NY, United States, 1:100), diluted in 2% BSA/PBS. Cnx was selected, because it acts together with ERp57 in a folding complex (Ni and Lee, 2007; Ryan et al., 2016). Next, the samples were rinsed with PBS (3 times for 5 min) and then incubated for 1 h at RT in the dark with Alexa Fluor^®^ 488-labelled donkey anti-rabbit IgG (A-21206, Invitrogen, West Grove, PA, United States, 1:500) in 2% BSA/PBS. After three washing steps using PBS, the silicone chambers of the IBIDI slides were removed and the samples were mounted with Fluoromount + DAPI (00–4959–52, Thermo Fisher, Waltham, MA, United States). A Leica DMi8 automated microscope (Leica Microsystems, Wetzlar, Germany) was used for image acquisition. Cells were encircled individually and the areas mean intensity was measured by ImageJ. All measured data points are presented in the corresponding figures.

### Nucleosome-ELISA for Quantification of Apoptotic Cell Death in C28/I2 Cells

To detect apoptosis, the Cell Death Detection ELISA^plus^ kit from Roche (11,774,425,001, Sigma Aldrich, Taufkirchen, Germany) was used. WT and KO cells were seeded in 400 µl DMEM complete into a 8-well IBIDI slide with a removable chamber at a density of 15,000 cells per well. After 4 h, the medium was changed to FCS-free DMEM complete and supplemented with 60 μg/ml β-aminopropionitrile fumarate, 25 μg/ml sodium ascorbate, 1 mM cysteine and 1 mM pyruvate (ABCP) +/- 10^–6^ M Tg, +/− 10^–11^ or 10^–15^ M estradiol. The cells were cultured in respective medium for 48 h at 37°C, 5% CO_2_ and 100% humidity. Apoptosis was detected according to the manufacturer’s instructions.

### Cartilage-Specific ERp57 Knockout Mice (ERp57 cKO)

ERp57 cKO mice (Linz et al., 2015) were kept in compliance with the German federal law for animal protection under the control of the North Rhine-Westphalia State Agency for Nature, Environment and Consumer Protection (LANUV, NRW, AZ 84-02.04.2017. A192) under pathogen-free conditions and supplied with food and water ad libitum in a 12-h light/dark cycle. To gain WT (ERp57 fl/fl) and ERp57 cKO (ERp57fl/fl-Col2a1-cre) littermates, homozygous ERp57 floxed Col2a1-cre-positive mice (ERp57fl/fl-Col2a1-cre) were mated with homozygous ERp57 floxed mice (ERp57 fl/fl).

### Preparation of Tissue Sections From Paraffin-Embedded Cartilage Tissues

To analyze ER stress, apoptosis and OA development in aging mice, WT and ERp57 cKO animals were sacrificed after 9, 12 or 18 months, and the knee samples were prepared for paraffin-embedding. First, tissues were fixed with 4% PFA in PBS at 4°C and immersed in decalcification solution containing 20% ethylenediaminetetraacetic acid (EDTA) and 6.6% (w/v) Tris. Under rotation the cartilage samples incubated for 2 weeks at RT, with the decalcification solution being changed every 2 days. Then, samples were dehydrated through a graded series of ethanol and isopropanol solutions and embedded in paraffin (Paraplast, X880.1, Roth, Karlsruhe, Germany). From paraffin blocks 4.5 µm thick sections were cut through the frontal plane of the knee joint with a rotation microtome AM355 (Microm, Wetzlar, Germany) and collected on glass slides.

### Immunofluorescence Analysis of Cartilage Samples

To evaluate the expression of the ER stress marker Cnx, sections of paraffin-embedded knee samples were deparaffinized and rehydrated. Before blocking of unspecific protein binding with 5% (w/v) BSA/PBS for 60 min at RT, the tissue sections were preincubated with 0.05% (w/v) protease XXIV in PBS for 10 min at 37°C and 0.1% (w/v) hyaluronidase in acetate buffer pH 6.0 for 90 min at 37°C. Then, the sections were rinsed with PBS and incubated overnight at 4°C with anti-Cnx antibody (ADI-SPA.860, Enzo Life Science, Farmingdale, NY, United States, 1:100) in 2% (w/v) BSA/PBS. The secondary antibody, Alexa Fluor^®^ 488 donkey anti-rabbit antibody (1:500, A-21206, Invitrogen, West Grove, PA, United States) was diluted in 2% a(w/v) BSA/PBS and supplied for 1 h at RT. The sections were rinsed in PBS and mounted with Fluoromount + DAPI (00–4959–52, Thermo Fisher, Waltham, MA, United States). Images were taken using a Leica DMi8 automated microscope and staining intensity was determined with the software ImageJ (Schneider et al., 2012).

### TUNEL Staining

Since ER stress in chondrocytes often induces apoptosis (Yang et al., 2005), deparaffinized and rehydrated knee sections were analyzed with the ApopTag^®^ Red *in situ* apoptosis detection kit from Millipore (Merck Chemicals, Gernsheim, Germany) according to the manufacturer’s instructions. After staining, the sections were mounted with Fluoromount + DAPI (00–4959–52, Thermo Fisher, Waltham, MA, United States) and images were taken with a Leica DMi8 automated microscope. The percentage of TUNEL-positive cells was quantified relative to the total number of DAPI-positive cells using ImageJ (Schneider et al., 2012).

### Histological Staining of Cartilage Samples

Sections of paraffin-embedded knee samples were deparaffinized and stained with 0.2% (w/v) Toluidine blue O (Serva, Heidelberg, Germany) in 0.2 M sodium acetate buffer pH 4.2 for 10 min at RT. After mounting with Entellan (Merck, Gernsheim, Germany), images were taken with a Nikon SMZ25 stereo microscope (Nikon Instruments, Tokyo, Japan).

### OA Scoring

OA severity was judged in frontal knee joints using the OARSI scoring system according to Glasson et al. (Glasson et al., 2010) with minor changes. Sulfated cartilage proteoglycans were stained with toluidine blue O. The scores include grades for cartilage degradation (0–6) plus grades for osteophyte formation (0–3) and are summed up to a maximal score of 9. See representative images for all stages of osteophyte formation (0 = without osteophyte formation, 1 = mild osteophyte formation, 2 = moderate osteophyte formation and 3 = severe osteophyte formation) in Supplementary Figure S1 of the supplemental material. Paraffin sections were cut through the whole joint and 2 independent scientists evaluated at least three sections in order to evaluate the entire joint in a blinded manner. The overall OA severity is reported as the maximal score of the medial and the lateral joint area. Additional hallmarks of OA were independently analyzed in (Rellmann et al., 2021b).

### Statistical Analysis

Evaluation of all samples was performed in a blinded manner. Damaged histological samples were excluded from evaluation. GraphPad Prism V.6.0 h (GraphPad Software Inc, La Jolla, CA, United States) was used for data presentation as scattered plots as means ± SD with a confidence interval of 95% and parametric, two-sided (Student *t*-test) tests with *p* < 0*.*05 determining the primary level of significance. The exact *p*-values are reported in the figure legends.

## Results

### Estradiol Inhibits ER Stress and ER Stress-Induced Apoptosis in C28/I2 Cells

**FIGURE 1:**
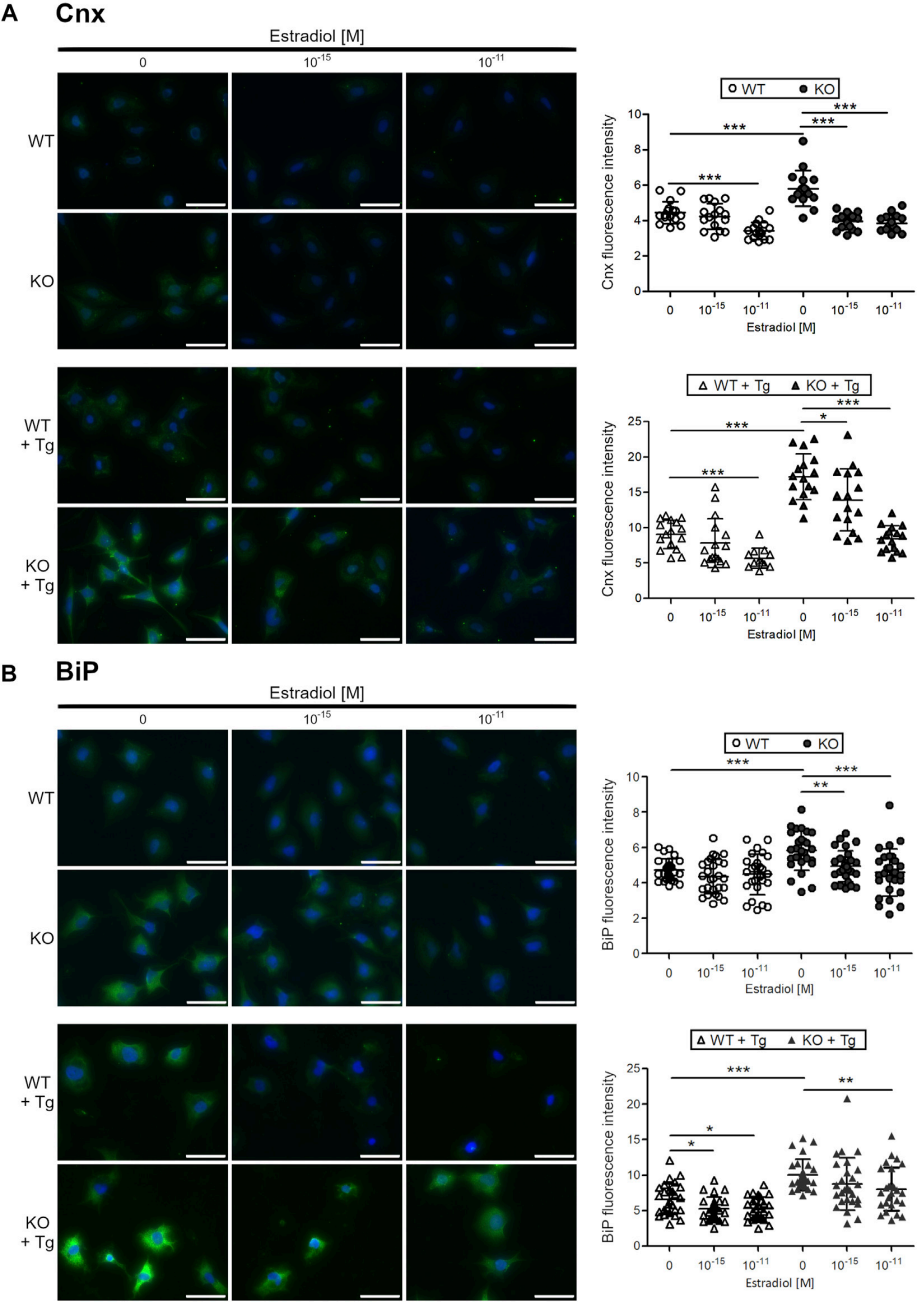
Estradiol reduces the protein expression of ER stress markers calnexin and BiP in C28/I2 WT and ERp57 KO chondrocytes *in vitro*. C28/I2 WT and ERp57 KO chondrocytes were cultured for 48 h in presence or absence of 1 µM of the ER stress inductor thapsigargin (Tg) with 0, 10^–15^, and 10^–11^ M estradiol. **(A)** Immunofluorescence staining of calnexin (Cnx) revealed a reduction of fluorescence intensity in WT cells by 10^–11^ M estradiol (*p* = 0.0000095). As expected, ERp57 KO cells showed a higher Cnx signal intensity than WT cells (*p* = 0.0000724), which was reduced by 10^–15^ M (*p* = 0.0000002) and 10^–11^ M (*p* = 0.0000001) estradiol. In presence of thapsigargin, Cnx expression is higher in both, WT and ERp57 KO chondrocytes with KO cells displaying even higher levels of Cnx fluorescence intensity than WT cells (*p* = 0.000000002). WT cells + Tg showed a reduced Cnx expression in presence of 10^–11^ M estradiol (*p* = 0.0000369). KO cells + Tg revealed a reduced Cnx expression by 10^–15^ M (*p* = 0.0214,753) and 10^–11^ M (*p* = 0.0000000002) estradiol. **(B)** Immunofluorescence staining of BiP showed that both estradiol concentrations were not effective to reduce BiP signals in WT cells. As expected, a higher BiP fluorescence intensity was measured in KO cells compared to WT cells (*p* = 0.0000460). This was decreased in presence of 10^–15^ M (*p* = 0.0019582) and 10^–11^ M (*p* = 0.0004781) estradiol. BiP signals in WT cells with thapsigargin treatment (WT + Tg) were slightly higher compared to untreated cells (WT) and were reduced in presence of 10^–15^ M (*p* = 0.0130,662) and 10^–11^ M (*p* = 0.0201,035) estradiol. KO cells + Tg showed a higher BiP fluorescence intensity than WT cells + Tg (*p* = 0.0000005) with a significant reduction by 10^–11^ M estradiol (*p* = 0.0076323). Scale bars = 50 µm. Cnx = Calnexin.

To test whether estradiol reduces ER stress in chondrocytes, C28/I2 WT and ERp57 KO chondrocytes were cultured in presence of estradiol. In humans, estradiol concentrations during the normal menstrual cycle range between 10^–11^ M bis 10^–9^ M (Claassen et al., 2011). For this study, different concentrations of estradiol were tested in the used cell culture systems in advance, orientating on physiological concentrations. See, Supplementary Figure S2. 10^–15^ and 10^–11^ M estradiol were chosen as concentrations appropriate for all *in vitro* experiments performed. ER stress levels were analyzed by immunofluorescence analysis of calnexin (Cnx, Figure 1A chaperone protein, induced by ER stress. In WT cells, a concentration of 10^–11^ M estradiol led to a significant decrease of Cnx fluorescence intensity. In KO cells, showing ER stress with higher Cnx levels due to loss of ERp57, both estradiol concentrations were highly effective in reducing Cnx fluorescence intensity down to WT levels. WT and ERp57 KO C28/I2 cells were additionally cultured in presence of the ER stress inductor Tg. Indeed, Cnx fluorescence intensity was increased in both, WT and KO cells cultured in presence of Tg. In appropriately cultured WT cells (WT + Tg) 10^–11^ M estradiol significantly reduced Cnx fluorescence intensity as a marker for ER stress. ERp57 KO cells cultured in presence of Tg displayed a higher Cnx signal compared to WT cells + Tg, which was efficiently reduced by both estradiol concentrations applied. Taken together, in all cell culture experiments of C28/I2 WT and ERp57 KO cells with or without Tg, estradiol effectively reduced ER stress as measured by the reduction of the fluorescence intensity of the ER stress marker Cnx.

A similar experimental setup was used to test the common ER stress marker protein BiP (Figure 1B). Immunofluorescence analysis revealed that in C28/I2 WT chondrocytes, BiP fluorescence intensity was not further reduced by neither of the two estradiol concentrations (10^–15^ and 10^–11^ M). However, C28/I2 ERp57 KO cells displayed a higher BiP fluorescence intensity and both estradiol concentrations significantly decreased the BiP signal. In WT and ERp57 KO cells cultivated in presence of Tg to increase ER stress levels, estradiol reduced the BiP fluorescence intensity significantly, especially with the concentration of 10^–11^ M. Since it was not possible to reduce BiP by estradiol in WT chondrocytes without ER stress induction by Tg, it is likely that a certain level of ER stress is necessary for estradiol to be effective against ER stress.

**FIGURE 2:**
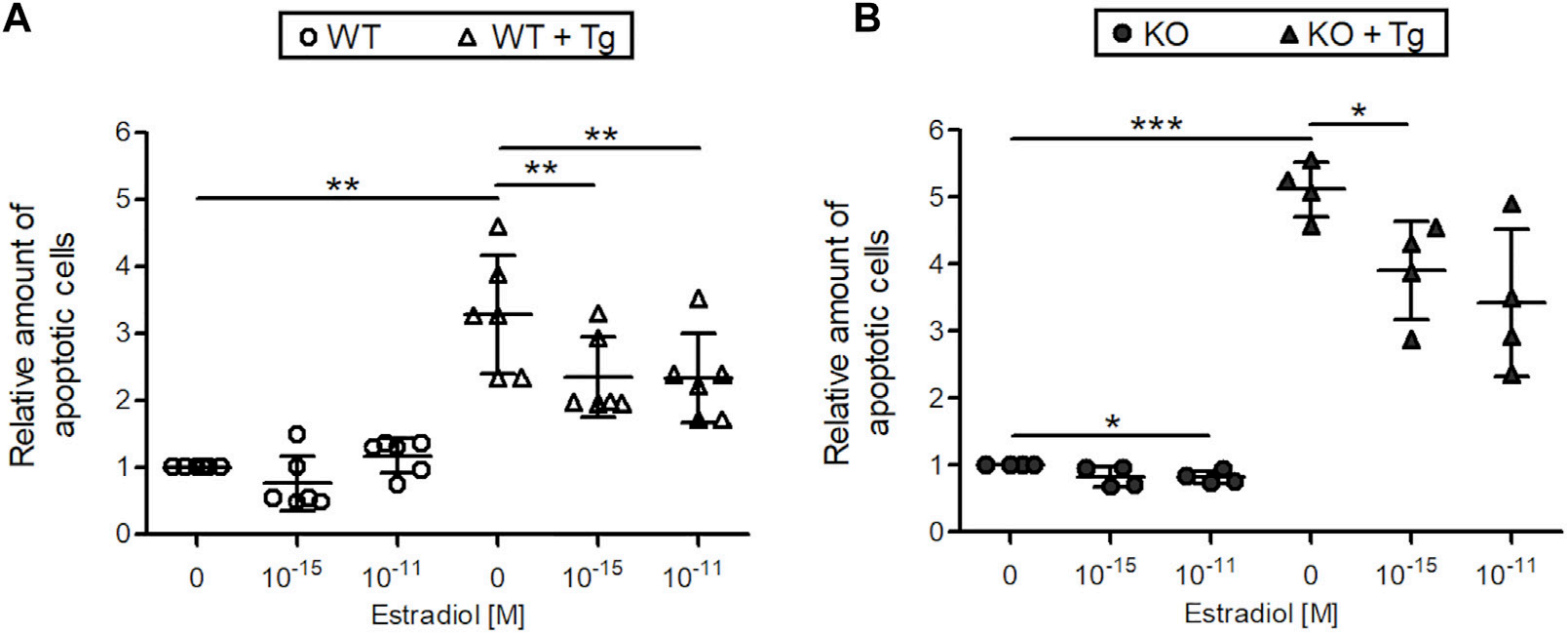
Estradiol reduces apoptosis in C28/I2 WT and ERp57 KO chondrocytes *in vitro*. C28/I2 WT **(A)** and ERp57 KO **(B)** chondrocytes were cultured for 48 h in presence or absence of 10 µM of the ER stress inductor thapsigargin (Tg) with 0, 10^–15^ and 10^–11^ M estradiol. Cell death ELISA revealed an increased number of apoptotic cells in WT (*p* = 0.0133944) and KO (*p* = 0.0002630) cell cultures by treatment with Tg. In WT cells + Tg apoptosis was reduced by 10^–15^ M (*p* = 0.0193,967) and 10^–11^ M (*p* = 0.0169453) estradiol. KO cells + Tg also showed a decreased number of apoptotic cells by 10^–15^ M estradiol (*p* = 0.0245,462). 10^–11^ M estradiol reduced apoptosis as well, but not significantly (*p* = 0.0789,528). Without ER stress induction by Tg estradiol was effective in KO cells with a concentration of 10^–11^ M (*p* = 0.0303279).

Next, the effect of 10^–11^ and 10^–15^ M estradiol on DNA strand breaks, a characteristic feature of apoptotic cells was measured by a cell death ELISA in C28/I2 WT and C28/I2 ERp57 KO cells (Figure 2). Without induction of ER stress with Tg, the number of apoptotic cells in both samples is very low. Nevertheless, apoptosis in C28/I2 ERp57 KO cells was reduced by 10^–11^ M estradiol. In WT and KO cells with high ER stress due to induction with Tg, the number of apoptotic cells was clearly increased and estradiol led to a dose-dependent inhibition of apoptosis. These results suggest that estradiol not only inhibits ER stress but also reduces ER stress-induced apoptosis in chondrocytes.

### Compared to Male Mice Articular Cartilage of Female Mice Displays a Slower Increase in Age-Related ER Stress

**FIGURE 3:**
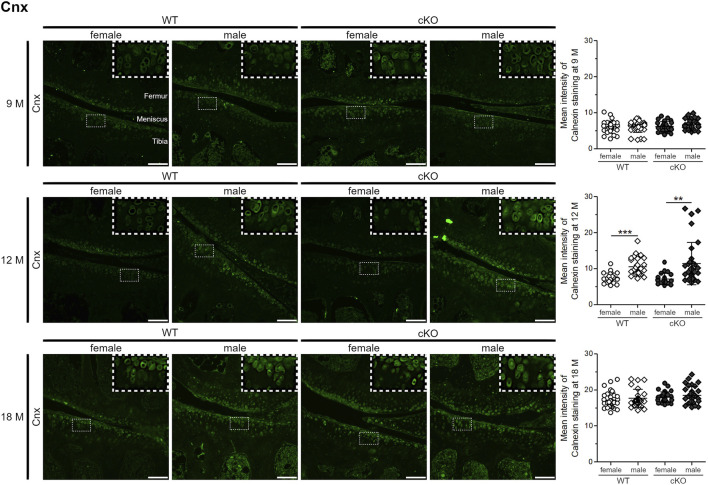
Age-related ER stress increases in articular chondrocytes at a slower rate in female mice than in male mice. 9-month-old knee cartilage of WT and ERp57 cKO mice reveals very low levels of the ER stress marker calnexin (Cnx) with no differences between genotypes and gender. At the age of 12 months, knee sections of male WT and ERp57 cKO mice disclose a higher immunofluorescence staining intensity of Cnx compared to female samples (male WT to female WT: *p* = 0.0000003; male cKO to female cKO: *p* = 0.0018094). At the age of 18 months, the overall calnexin staining intensity increased, but is comparable between the analyzed groups. Representative image sections were marked and shown 3-fold enlarged in the upper right corner. Scale bars = 100 µm. M = months, Cnx = Calnexin.

ER stress levels were tested *in vivo* in female and male WT and ERp57 cKO mice (Figure 3). ER stress marker Cnx was used to analyze ER stress levels in the articular knee cartilage at the age of 9, 12, and 18 months. In 9-month-old animals, Cnx staining intensity is quite low and no differences between the genotypes or sexes were observed. This changes at the age of 12 months. One-year-old male WT and ERp57 cKO mice display a rise in the staining intensity of the ER stress marker Cnx compared to 9-month-old mice, whereas the Cnx levels of female littermates remain low and stay constant. At this age, differences in ER stress appears to be more dependent on gender than on genotype. At the age of 18 months, a balance of these differences between the four groups was observed. However, at this age, Cnx staining intensity was much higher in all genotypes and sexes than at 9 and 12 months, suggesting an increase in ER stress in articular cartilage with aging in both sexes, but increasing at different rates depending on sex.

### Female ERp57 cKO Mice Develop less Apoptotic Cells During Ageing Compared to Male Mice

**FIGURE 4:**
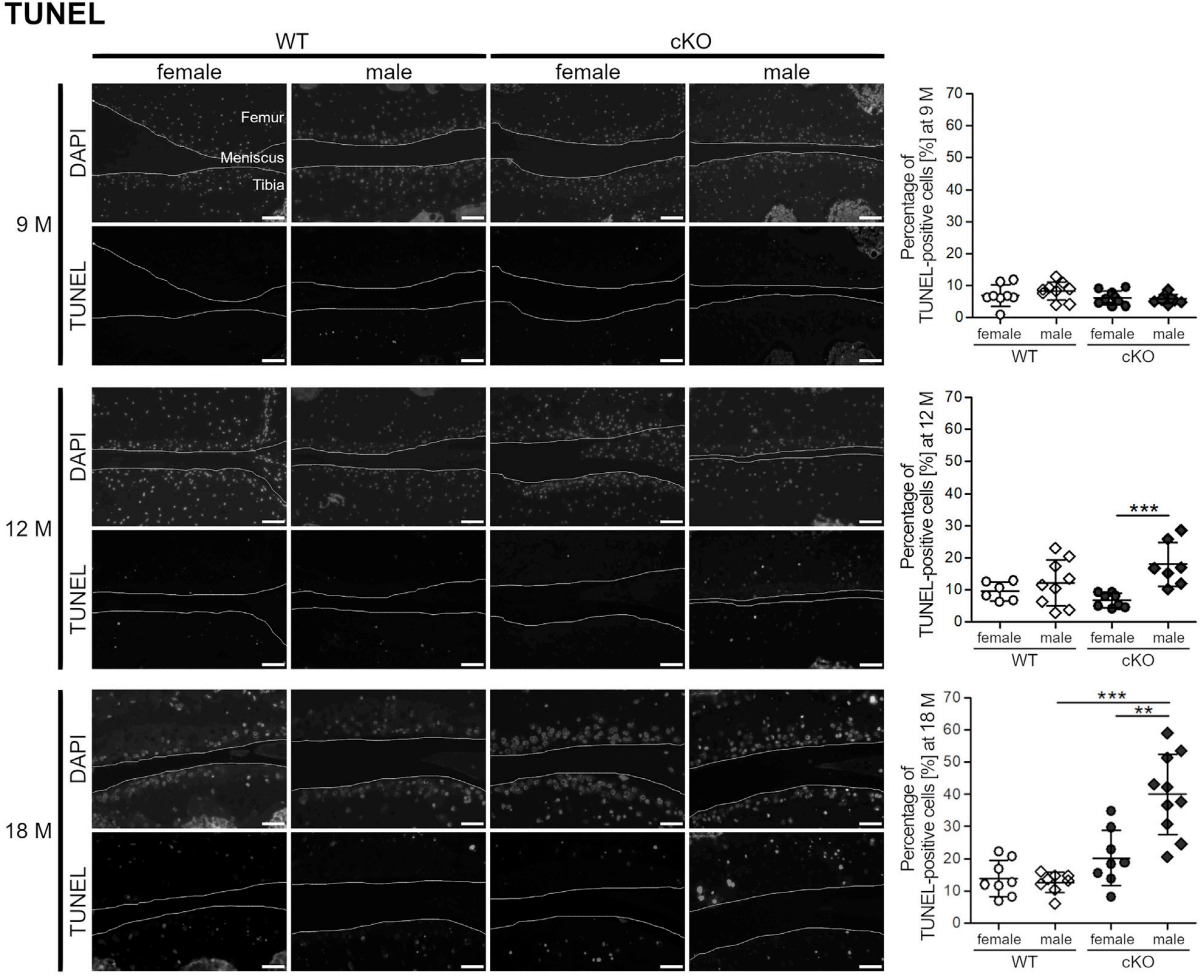
Female mice develop less apoptotic cells during ageing compared to male mice. TUNEL staining reveals a higher percentage of apoptotic cells in the knee sections of 12 months old male ERp57 cKO mice, while female mice remain on a low level (*p* = 0.00069). This difference is even increased at the age of 18 months (*p* = 0.00161,699). Then, male cKO mice additionally have significantly higher numbers of apoptotic cells than male WT mice (*p* = 0.00001840). Joint surfaces are highlighted. Scale bars = 50 μm, M = months.

Articular cartilage of 9-, 12- and 18-month-old female and male WT and cKO mice was analyzed by TUNEL staining (Figure 4). At the age of 9 months, apoptotic cell death was low. Less than 10% of chondrocytes displayed DNA strand breaks and no difference between sexes and genotypes was observed. 12-month-old mice displayed a significantly higher percentage of apoptotic cells in the male ERp57 cKO group, which correlates with the higher ER stress analyzed by Cnx staining. At the age of 18 months, this difference was even higher. Female and male WT mice, as well as female cKO mice displayed less than 20% of apoptotic cells compared to an average of 40% of apoptotic cells in male cKO mice. All in all, high ER stress in the male genotype leads to increased apoptosis of articular chondrocytes, while female mice seem to be protected from high ER stress and remain at a very low level of apoptosis.

### Female Mice Develop OA With a Lower Degree of Severity Compared to Male Animals

**FIGURE 5:**
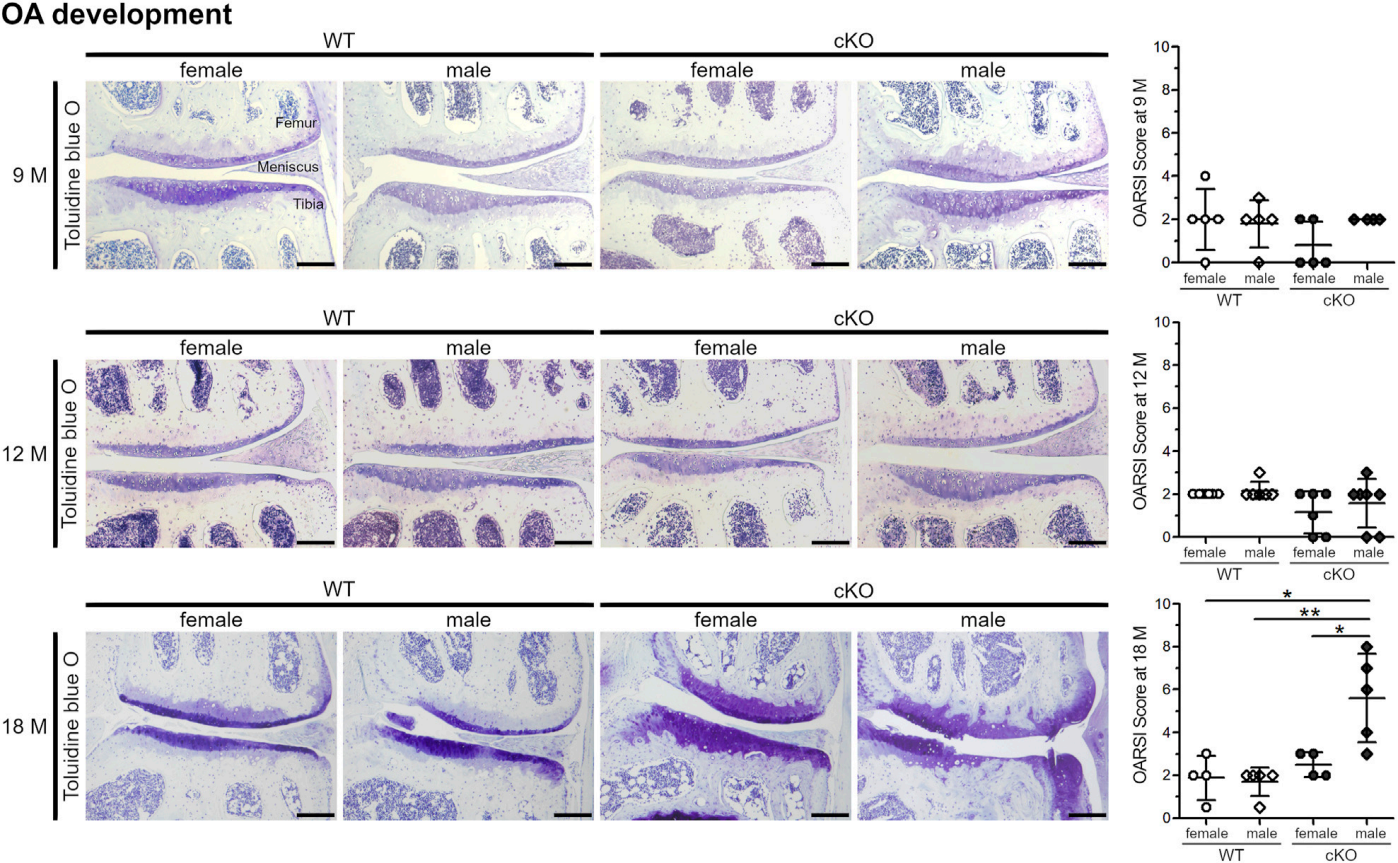
Male ERp57cKO mice develop strong signs of OA during ageing. At the age of 18 months, OARSI scoring of toluidine blue O-stained knee sections reveals an increase of cartilage degradation, including osteophyte formation in male cKO mice, but not in female cKO mice (*p* = 0.0241313), nor female WT (*p* = 0.01398420) or male WT mice (*p* = 0.00394253). At earlier timepoints (9 and 12 M), no difference between the sexes or genotypes is observed. Scale Bar = 100 μm, M = months.

Apoptosis of articular chondrocytes is known to precede cartilage degradation and OA development (Hwang and Kim, 2015; Rellmann et al., 2021b). Therefore, the articular cartilage of 9-, 12- and 18-month-old mice of female and male WT and cKO mice was analyzed for cartilage degeneration by toluidine blue O staining and a subsequent evaluation of cartilage degradation by OARSI scoring (Figure 5). At the age of 9 and 12 months, the articular cartilage in all four analyzed groups looked healthy and smooth. In 18-month-old mice, a severe cartilage breakdown as well as osteophyte formation with OARSI-Scores of 4–8 was observed in male cKO mice, while the cartilage of WT mice and female cKO mice was almost unaffected (OARSI-Score around 2). Our results suggest, that high ER stress inducing a high percentage of apoptotic cells induces osteoarthritic cartilage degeneration in male cKO mice, while female mice seem to be protected. Estradiol, that reduces ER stress and apoptosis of chondrocytes *in vitro*, is likely to also reduce ER stress-induced apoptosis *in vivo* and this explains why female mice develop less severe age-related osteoarthritic cartilage degeneration than male mice.

## Discussion

The present study reveals that knee joints of female ERp57 cKO mice exhibit less osteoarthritic cartilage degradation with increasing age compared to male mice. Interestingly, this is associated with a lower ER stress in chondrocytes, which results in a reduced number of apoptotic cartilage cells. Therefore, the role of estradiol in ER stress-induced apoptosis of chondrocytes was extensively evaluated *in vitro*. These cell culture experiments showed physiological concentrations of the female sex hormone estradiol to reduce ER stress and ER stress-induced apoptosis in chondrocytes, thus minimizing critical events contributing to osteoarthritic cartilage degeneration.

Healthy articular cartilage consists of large volumes of ECM, produced and maintained by sparsely distributed chondrocytes within. During synthesis, ECM proteins are translated into the ER to be glycosylated and folded. An overload of the ER stacks with unfolded or misfolded ECM proteins thus is critical and leads to ER stress with subsequent induction of the UPR, lowering the translation of essential matrix proteins. AFM analysis clearly revealed that the compressive stiffness of cartilage collagens (mainly collagen II) and proteoglycans (mainly aggrecan) is reduced as a consequence of the reduced ECM protein production under ER stress (Rellmann et al., 2021b).

A compromised ECM protein production decreases a proper cartilage function and was shown to contribute to the pathogenesis of OA (Mueller and Tuan, 2011).

If ER stress is severe or lasts for a long period of time, the UPR initiates apoptotic cell death (Rellmann et al., 2021a), also a hallmark of OA (Uehara et al., 2014). Both ER stress models used, C28/I2 ERp57 KO cells and cartilage specific ERp57 KO mice, show many characteristics of ER stress, e.g., all three UPR signaling pathways are active and apoptosis of chondrocytes is initiated (Linz et al., 2015; Rellmann and Dreier, 2018; Rellmann et al., 2019; Rellmann et al., 2021b). And, as in humans, osteoarthritic cartilage degeneration in ERp57 cKO mice was shown to increase with age (Rellmann et al., 2021b), most likely due to higher ER stress levels through loss of ERp57 and an age-related decline in ER stress compensation resulting in induction of chondrocyte apoptosis (Brown and Naidoo, 2012).

A comparison of the severity of ER stress-induced cartilage degeneration in male and female ERp57 cKO mice reveals a clear sex-specific variation, with male mice being affected much more severely than female animals. Here, this difference was attributed to the influence of female sex hormones, which prevent ER stress-induced apoptosis usually associated with OA cartilage degradation. This should be confirmed in ovariectomized WT and ERp57-cKO mice with or without application of defined amounts of estradiol in future experiments, because other possibilities exist. For example, different mechanical effects could occur because male mice having more weight than females. In addition, differences in bone structure and bone mass in female and male mice are described, or divergent effects of autophagy and inflammation. These and other gender-specific aspects in OA are described in (Contartese et al., 2020).

Protective effects of estradiol in OA development were previously observed in several studies (Ma et al., 2007; Roman-Blas et al., 2009; Tanamas et al., 2011; Imgenberg et al., 2013), but this has never been attributed to an estradiol-induced reduction of ER stress-induced apoptosis in cartilage cells. For example, estradiol was noticed to inhibit mechanically-induced apoptosis in chondrocytes (Imgenberg et al., 2013) and to promote autophagy via ERK-mTOR signaling (Ge et al., 2019), which also reduces the protein load in the ER and thus has the potential to ameliorate ER stress. However, it remains unclear whether ER stress is involved in the described changes in the mouse models used. The inhibitory effect of estradiol on cell shrinkage as an apoptotic event in cultured rabbit articular chondrocytes (Kumagai et al., 2012) was not attributed to ER stress either.

In chondrocytes, estradiol signals via the classical estrogen receptors alpha (ERα) and beta (ERβ) (Ushiyama et al., 1999). Interestingly, polymorphisms of estrogen receptor genes have been associated with a higher risk of generalized osteoarthritis OA (Ushiyama et al., 1998). In addition, the nonclassical G-protein coupled estrogen receptor (GPER/GPR30), mediating rapid cellular responses, including activation of ion channels, kinases and signaling pathways (Fan et al., 2018), is present in cartilage cells (Chagin and Savendahl, 2007; Engdahl et al., 2010). Interestingly, some rapid estradiol effects in cartilage cells occurred only in female animals and were described as cell maturation dependent (Schwartz et al., 1996). However, if these are mediated via ERα and ERβ or via G-protein coupled estrogen receptor signaling remains open, since there is evidence for both options (Chou et al., 2021; Joshua Cohen et al., 2021). Whether ERα, ERβ or GPR30 or all synergistically are involved in mediating the observed estradiol-dependent reduction in ER stress-induced apoptosis in cartilage cells of female mice remains to be determined in future studies.

It is also significant that some chondrocytes can locally produce estradiol. Growth plate chondrocytes have been shown to generate and secrete estradiol, which protects cells from apoptosis during longitudinal bone growth (Chagin et al., 2006). Whether articular cartilage cells are also capable of producing estradiol and whether this estradiol inhibits ER stress to reduce chondrocytic cell death in joints has not yet been studied in detail. This is however, a conceivable mechanism since some features of osteoarthritic cartilage degeneration resemble those of endochondral ossification (Dreier, 2010).

Taken together, the cross-talk between female sex hormones and ER stress-induced apoptosis is a critical event in osteoarthritic cartilage degeneration, which would offer novel targets for therapeutic interventions. Estrogen effects were already evaluated in preclinical studies and clinical trials, but were partially inconclusive (Sniekers et al., 2008). However, almost all studies assessing selective estrogen receptor modulators (SERMs) have obtained beneficial effects for OA patients with a relatively safety and tolerability profiles (Xiao et al., 2016). For example, tamoxifene (Rosner et al., 1982; Colombo et al., 1983; Rosner et al., 1983), CHPPPC (Hoegh-Andersen et al., 2004) and levormeloxifene (Christgau et al., 2004) have been shown to reduce cartilage damage in animal models of OA. Nevertheless, further research is indispensable to determine the most beneficial SERMs to treat OA, as there is a wide range of different agents with different chemical structures and biological functions (Xiao et al., 2016). Besides estrogen or SERMs, ER stress modulators could be valuable drugs to prevent osteoarthritic cartilage degeneration. The reduction of ER stress, e.g. by application of chemical chaperones or stimulation of autophagy, or the inhibition of apoptosis-promoting UPR signaling pathways could be successful future strategies for treating OA patients, which are worth investigating in detail (Rellmann et al., 2021a).

## Conclusion

The female sex hormone estradiol can reduce ER stress and ER stress-induced apoptosis in articular chondrocytes, thus minimizing critical events favoring osteoarthritic cartilage degeneration. These results help to reassess and explore novel targets for therapeutic interventions in OA.

## Data Availability

The raw data supporting the conclusion of this article will be made available by the authors, without undue reservation.

## References

[B1] Brown M. K. Naidoo N. (2012). The Endoplasmic Reticulum Stress Response in Aging and Age-Related Diseases. Front. Physio. 3, 263. 10.3389/fphys.2012.00263 PMC3429039 22934019

[B2] Chagin A. S. Chrysis D. Takigawa M. Ritzen E. M. Sävendahl L. (2006). Locally Produced Estrogen Promotes Fetal Rat Metatarsal Bone Growth; an Effect Mediated through Increased Chondrocyte Proliferation and Decreased Apoptosis. J. Endocrinol. 188 (2), 193–203. 10.1677/joe.1.06364 16461546

[B3] Chagin A. S. Sävendahl L. (2007). GPR30 Estrogen Receptor Expression in the Growth Plate Declines as Puberty Progresses. J. Clin. Endocrinol. Metab. 92 (12), 4873–4877. 10.1210/jc.2007-0814 17878253

[B4] Charlier E. Relic B. Deroyer C. Malaise O. Neuville S. Collée J. (2016). Insights on Molecular Mechanisms of Chondrocytes Death in Osteoarthritis. Ijms 17 (12), 2146. 10.3390/ijms17122146 PMC5187946 27999417

[B5] Chou Y.-S. Chuang S.-C. Chen C.-H. Ho M.-L. Chang J.-K. (2021). G-Protein-Coupled Estrogen Receptor-1 Positively Regulates the Growth Plate Chondrocyte Proliferation in Female Pubertal Mice. Front. Cell Dev. Biol. 9, 710664. 10.3389/fcell.2021.710664 34490260 PMC8417792

[B6] Christgau S. Tankó L. B. Cloos P. A. C. Mouritzen U. Christiansen C. Delaissé J.-M. (2004). Suppression of Elevated Cartilage Turnover in Postmenopausal Women and in Ovariectomized Rats by Estrogen and a Selective Estrogen-Receptor Modulator (SERM). Menopause 11 (5), 508–518. 10.1097/01.wcb.0000121484.18437.98 15356403

[B7] Claassen H. Schicht M. Brandt J. Reuse K. Schädlich R. Goldring M. B. (2011). C-28/I2 and T/C-28a2 Chondrocytes as Well as Human Primary Articular Chondrocytes Express Sex Hormone and Insulin Receptors-Useful Cells in Study of Cartilage Metabolism. Ann. Anat. - Anatomischer Anzeiger 193 (1), 23–29. 10.1016/j.aanat.2010.09.005 20971625 PMC3937963

[B8] Coe H. Michalak M. (2010). ERp57, a Multifunctional Endoplasmic Reticulum Resident Oxidoreductase. Int. J. Biochem. Cell Biol. 42 (6), 796–799. 10.1016/j.biocel.2010.01.009 20079872

[B9] Colombo C. Butler M. Hickman L. Selwyn M. Chart J. Steinetz B. (1983). A New Model of Osteoarthritis in Rabbits II. Evaluation of Anti-osteoarthritic Effects of Selected Antirheumatic Drugs Administered Systemically. Arthritis. Rheumatism 26 (9), 1132–1139. 10.1002/art.1780260911 6688529

[B10] Contartese D. Tschon M. De Mattei M. Fini M. (2020). Sex Specific Determinants in Osteoarthritis: A Systematic Review of Preclinical Studies. Ijms 21 (10), 3696. 10.3390/ijms21103696 PMC7279293 32456298

[B11] Denmeade S. R. Isaacs J. T. (2005). The SERCA Pump as a Therapeutic Target: Making a "smart Bomb" for Prostate Cancer. Cancer Biol. Ther. 4 (1), 21–29. 10.4161/cbt.4.1.1505 15662118

[B12] Dreier R. (2010). Hypertrophic Differentiation of Chondrocytes in Osteoarthritis: the Developmental Aspect of Degenerative Joint Disorders. Arthritis Res. Ther. 12 (5), 216. 10.1186/ar3117 20959023 PMC2990991

[B13] Engdahl C. Jochems C. Windahl S. H. Bã¶rjesson A. E. Ohlsson C. Carlsten H. (2010). Signaling via Estrogen Receptor Alpha, and Not Estrogen Receptor Beta or Gpr30, Ameliorates Collagen-Induced Arthritis and Immune-Associated Bone Loss. Arthritis Rheum. 62 (2), NA. 10.1002/art.25055 20112355

[B14] Eun Y. Yoo J. E. Han K. Kim D. Lee K. N. Lee J. (2022). Female Reproductive Factors and Risk of Joint Replacement Arthroplasty of the Knee and Hip Due to Osteoarthritis in Postmenopausal Women: a Nationwide Cohort Study of 1.13 Million Women. Osteoarthr. Cartil. 30 (1), 69–80. 10.1016/j.joca.2021.10.012 34774788

[B15] Fan D.-x. Yang X.-h. Li Y.-n. Guo L. (2018). 17β-Estradiol on the Expression of G-Protein Coupled Estrogen Receptor (GPER/GPR30) Mitophagy, and the PI3K/Akt Signaling Pathway in ATDC5 Chondrocytes *In Vitro* . Med. Sci. Monit. 24, 1936–1947. 10.12659/msm.909365 29608013 PMC5898603

[B16] Finger F. Schörle C. Zien A. Gebhard P. Goldring M. B. Aigner T. (2003). Molecular Phenotyping of Human Chondrocyte Cell Lines T/C-28a2, T/C-28a4, and C-28/I2. Arthritis & Rheumatism 48 (12), 3395–3403. 10.1002/art.11341 14673991

[B17] Fuchs J. K. Ronny Z. Scheidt-Nave C. (2017). 12-Monats-Prävalenz von Arthrose in Deutschland. J. Health Monit. 2 **,** 55–60. 10.17886/RKI-GBE-2017-054

[B18] Ge Y. Zhou S. Li Y. Wang Z. Chen S. Xia T. (2019). Estrogen Prevents Articular Cartilage Destruction in a Mouse Model of AMPK Deficiency via ERK-mTOR Pathway. Ann. Transl. Med. 7 (14), 336. 10.21037/atm.2019.06.77 31475206 PMC6694256

[B19] Glasson S. S. Chambers M. G. Van Den Berg W. B. Little C. B. (2010). The OARSI Histopathology Initiative - Recommendations for Histological Assessments of Osteoarthritis in the Mouse. Osteoarthr. Cartil. 18 (Suppl. 3), S17–S23. 10.1016/j.joca.2010.05.025 20864019

[B20] Goldring M. B. Goldring S. R. (2007). Osteoarthritis. J. Cell. Physiol. 213 (3), 626–634. 10.1002/jcp.21258 17786965

[B21] Hettinghouse A. Liu R. Liu C.-j. (2018). Multifunctional Molecule ERp57: From Cancer to Neurodegenerative Diseases. Pharmacol. Ther. 181, 34–48. 10.1016/j.pharmthera.2017.07.011 28723413 PMC5743601

[B22] Hetz C. (2012). The Unfolded Protein Response: Controlling Cell Fate Decisions under ER Stress and beyond. Nat. Rev. Mol. Cell Biol. 13 (2), 89–102. 10.1038/nrm3270 22251901

[B23] Hetz C. Zhang K. Kaufman R. J. (2020). Mechanisms, Regulation and Functions of the Unfolded Protein Response. Nat. Rev. Mol. Cell Biol. 21 (8), 421–438. 10.1038/s41580-020-0250-z 32457508 PMC8867924

[B24] Høegh-Andersen P. Tankó L. B. Andersen T. L. Lundberg C. V. Mo J. A. Heegaard A.-M. (2004). Ovariectomized Rats as a Model of Postmenopausal Osteoarthritis: Validation and Application. Arthritis Res. Ther. 6 (2), R169–R180. 10.1186/ar1152 15059281 PMC400436

[B25] Hwang H. Kim H. (2015). Chondrocyte Apoptosis in the Pathogenesis of Osteoarthritis. Ijms 16 (11), 26035–26054. 10.3390/ijms161125943 26528972 PMC4661802

[B26] Imgenberg J. Rolauffs B. Grodzinsky A. J. Schünke M. Kurz B. (2013). Estrogen Reduces Mechanical Injury-Related Cell Death and Proteoglycan Degradation in Mature Articular Cartilage Independent of the Presence of the Superficial Zone Tissue. Osteoarthr. Cartil. 21 (11), 1738–1745. 10.1016/j.joca.2013.07.007 23863610

[B27] Joshua Cohen D. ElBaradie K. Boyan B. D. Schwartz Z. (2021). Sex-specific Effects of 17β-Estradiol and Dihydrotestosterone (DHT) on Growth Plate Chondrocytes Are Dependent on Both ERα and ERβ and Require Palmitoylation to Translocate the Receptors to the Plasma Membrane. Biochimica Biophysica Acta (BBA) - Mol. Cell Biol. Lipids 1866 (12), 159028. 10.1016/j.bbalip.2021.159028 34416391

[B28] Kumagai K. Imai S. Toyoda F. Okumura N. Isoya E. Matsuura H. (2012). 17β-Oestradiol Inhibits Doxorubicin-Induced Apoptosis via Block of the Volume-Sensitive Cl- Current in Rabbit Articular Chondrocytes. Br. J. Pharmacol. 166 (2), 702–720. 10.1111/j.1476-5381.2011.01802.x 22142024 PMC3417499

[B29] Linz A. Knieper Y. Gronau T. Hansen U. Aszodi A. Garbi N. (2015). ER Stress during the Pubertal Growth Spurt Results in Impaired Long-Bone Growth in Chondrocyte-specific ERp57 Knockout Mice. J. Bone Min. Res. 30 (8), 1481–1493. 10.1002/jbmr.2484 25704664

[B30] Ma H.-L. Blanchet T. J. Peluso D. Hopkins B. Morris E. A. Glasson S. S. (2007). Osteoarthritis Severity Is Sex Dependent in a Surgical Mouse Model. Osteoarthr. Cartil. 15 (6), 695–700. 10.1016/j.joca.2006.11.005 17207643

[B31] Mueller M. B. Tuan R. S. (2011). Anabolic/Catabolic Balance in Pathogenesis of Osteoarthritis: Identifying Molecular Targets. PM&R 3 (6 Suppl. 1), S3–S11. 10.1016/j.pmrj.2011.05.009 21703577

[B32] Ni M. Lee A. S. (2007). ER Chaperones in Mammalian Development and Human Diseases. FEBS Lett. 581 (19), 3641–3651. 10.1016/j.febslet.2007.04.045 17481612 PMC2040386

[B33] Nugent A. E. Speicher D. M. Gradisar I. McBurney D. L. Baraga A. Doane K. J. (2009). Advanced Osteoarthritis in Humans Is Associated with Altered Collagen VI Expression and Upregulation of ER-Stress Markers Grp78 and Bag-1. J. Histochem Cytochem. 57 (10), 923–931. 10.1369/jhc.2009.953893 19546472 PMC2746726

[B34] Rellmann Y. Dreier R. (2018). Different Forms of ER Stress in Chondrocytes Result in Short Stature Disorders and Degenerative Cartilage Diseases: New Insights by Cartilage-specific ERp57 Knockout Mice. Oxidative Med. Cell. Longev. 2018, 1–14. 10.1155/2018/8421394 PMC6311764 30647818

[B35] Rellmann Y. Eidhof E. Dreier R. (2021a). Review: ER Stress-Induced Cell Death in Osteoarthritic Cartilage. Cell. Signal. 78, 109880. 10.1016/j.cellsig.2020.109880 33307190

[B36] Rellmann Y. Eidhof E. Hansen U. Fleischhauer L. Vogel J. Clausen-Schaumann H. (2021b). ER Stress in ERp57 Knockout Knee Joint Chondrocytes Induces Osteoarthritic Cartilage Degradation and Osteophyte Formation. Ijms 23 (1), 182. 10.3390/ijms23010182 35008608 PMC8745280

[B37] Rellmann Y. Gronau I. Hansen U. Dreier R. (2019). 4-Phenylbutyric Acid Reduces Endoplasmic Reticulum Stress in Chondrocytes that Is Caused by Loss of the Protein Disulfide Isomerase ERp57. Oxidative Med. Cell. Longev. 2019, 1–12. 10.1155/2019/6404035 PMC6875354 31781343

[B38] Roman-Blas J. A. Castañeda S. Largo R. Herrero-Beaumont G. (2009). Osteoarthritis Associated with Estrogen Deficiency. Arthritis Res. Ther. 11 (5), 241. 10.1186/ar2791 19804619 PMC2787275

[B39] Rosner I. A. Malemud C. J. Goldberg V. M. Papay R. S. Getzy L. Moskowitz R. W. (1982). Pathologic and Metabolic Responses of Experimental Osteoarthritis to Estradiol and an Estradiol Antagonist. Clin. Orthop. Relat. Res. 171, 280–286. 10.1097/00003086-198211000-00047 7140079

[B40] Rosner I. A. Malemud C. J. Hassid A. I. Goldberg V. M. Boja B. A. Moskowitz R. W. (1983). Estradiol and Tamoxifen Stimulation of Lapine Articular Chondrocyte Prostaglandin Synthesis. Prostaglandins 26 (1), 123–138. 10.1016/0090-6980(83)90080-1 6635209

[B41] Ryan D. Carberry S. Murphy Á. C. Lindner A. U. Fay J. Hector S. (2016). Calnexin, an ER-Induced Protein, Is a Prognostic Marker and Potential Therapeutic Target in Colorectal Cancer. J. Transl. Med. 14 (1), 196. 10.1186/s12967-016-0948-z 27369741 PMC4930591

[B42] Schneider C. A. Rasband W. S. Eliceiri K. W. (2012). NIH Image to ImageJ: 25 Years of Image Analysis. Nat. Methods 9 (7), 671–675. 10.1038/nmeth.2089 22930834 PMC5554542

[B43] Schwartz Z. Gates P. A. Nasatzky E. Sylvia V. L. Mendez J. Dean D. D. (1996). Effect of 17β-Estradiol on Chondrocyte Membrane Fluidity and Phospholipid Metabolism Is Membrane-specific, Sex-specific, and Cell Maturation-dependent. Biochimica Biophysica Acta. 1282 (1), 1–10. 10.1016/0005-2736(96)00019-3 8679644

[B44] Sniekers Y. H. Weinans H. Bierma-Zeinstra S. M. van Leeuwen J. P. T. M. van Osch G. J. V. M. (2008). Animal Models for Osteoarthritis: the Effect of Ovariectomy and Estrogen Treatment - a Systematic Approach. Osteoarthr. Cartil. 16 (5), 533–541. 10.1016/j.joca.2008.01.002 18280756

[B45] Srikanth V. K. Fryer J. L. Zhai G. Winzenberg T. M. Hosmer D. Jones G. (2005). A Meta-Analysis of Sex Differences Prevalence, Incidence and Severity of Osteoarthritis. Osteoarthr. Cartil. 13 (9), 769–781. 10.1016/j.joca.2005.04.014 15978850

[B46] Takada K. Hirose J. Senba K. Yamabe S. Oike Y. Gotoh T. (2011). Enhanced Apoptotic and Reduced Protective Response in Chondrocytes Following Endoplasmic Reticulum Stress in Osteoarthritic Cartilage. Int. J. Exp. Pathol. 92 (4), 232–242. 10.1111/j.1365-2613.2010.00758.x 21294793 PMC3144511

[B47] Tanamas S. K. Wijethilake P. Wluka A. E. Davies-Tuck M. L. Urquhart D. M. Wang Y. (2011). Sex Hormones and Structural Changes in Osteoarthritis: a Systematic Review. Maturitas 69 (2), 141–156. 10.1016/j.maturitas.2011.03.019 21481553

[B48] Tsang K. Y. Chan D. Cheslett D. Chan W. C. W. So C. L. Melhado I. G. (2007). Surviving Endoplasmic Reticulum Stress Is Coupled to Altered Chondrocyte Differentiation and Function. PLoS. Biol. 5 (3), e441544–9173. 10.1371/journal.pbio.0050044 PMC1820825 17298185

[B49] Uehara Y. Hirose J. Yamabe S. Okamoto N. Okada T. Oyadomari S. (2014). Endoplasmic Reticulum Stress-Induced Apoptosis Contributes to Articular Cartilage Degeneration via C/EBP Homologous Protein. Osteoarthr. Cartil. 22 (7), 1007–1017. 10.1016/j.joca.2014.04.025 24795271

[B50] Ushiyama T. Ueyama H. Inoue K. Nishioka J. Ohkubo I. Hukuda S. (1998). Estrogen Receptor Gene Polymorphism and Generalized Osteoarthritis. J. Rheumatol. 25 (1), 134 9458216

[B51] Ushiyama T. Ueyama H. Inoue K. Ohkubo I. Hukuda S. (1999). Expression of Genes for Estrogen Receptors α and β in Human Articular Chondrocytes. Osteoarthr. Cartil. 7 (6), 560–566. 10.1053/joca.1999.0260 10558854

[B52] Wilson M. G. Michet C. J. Jr. Ilstrup D. M. Melton Iii L. J. 3rd (1990). Idiopathic Symptomatic Osteoarthritis of the Hip and Knee: a Population-Based Incidence Study. Mayo Clin. Proc. 65 (9), 1214–1221. 10.1016/s0025-6196(12)62745-1 2402161

[B53] Xiao Y.-P. Tian F.-M. Dai M.-W. Wang W.-Y. Shao L.-T. Zhang L. (2016). Are Estrogen-Related Drugs New Alternatives for the Management of Osteoarthritis? Arthritis Res. Ther. 18, 151. 10.1186/s13075-016-1045-7 27352621 PMC4924302

[B54] Yang L. Carlson S. G. McBurney D. Horton W. E. Jr. (2005). Multiple Signals Induce Endoplasmic Reticulum Stress in Both Primary and Immortalized Chondrocytes Resulting in Loss of Differentiation, Impaired Cell Growth, and Apoptosis. J. Biol. Chem. 280 (35), 31156–31165. 10.1074/jbc.M501069200 16000304

