# Supplementary figures and images for: Estradiol Inhibits ER Stress-Induced Apoptosis in Chondrocytes and Contributes to a Reduced Osteoarthritic Cartilage Degeneration in Female Mice
Source: Front Cell Dev Biol. 2022 May 20;10:913118. doi: 10.3389/fcell.2022.913118 (PMC9163336; doi:10.3389/fcell.2022.913118)

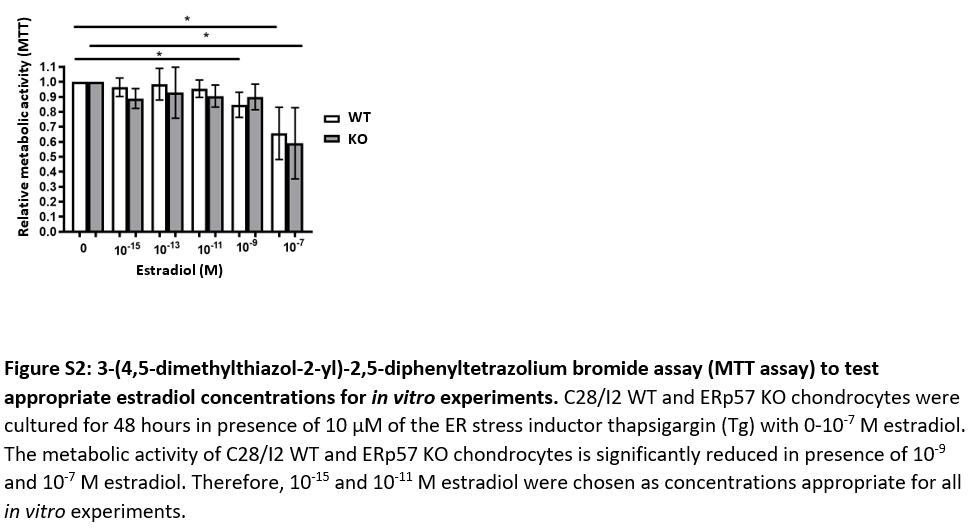

Supplement: Supplementary file 1 [file Image2.tif]

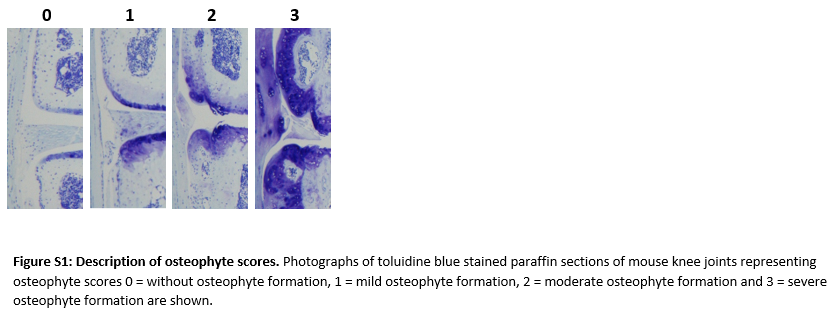

Supplement: Supplementary file 2 [file Image1.tif]
